# Self-reported vision impairment and incident prefrailty and frailty in English community-dwelling older adults: findings from a 4-year follow-up study

**DOI:** 10.1136/jech-2017-209207

**Published:** 2017-08-10

**Authors:** Ann E M Liljas, Livia A Carvalho, Efstathios Papachristou, Cesar De Oliveira, S Goya Wannamethee, Sheena E Ramsay, Kate R Walters

**Affiliations:** 1 Department of Primary Care and Population Health, University College London, London, UK; 2 Department of Clinical Pharmacology, Queen Mary University of London, London, UK; 3 Department of Epidemiology and Public Health, University College London, London, UK; 4 Institute of Health and Society, Newcastle University, Newcastle upon Tyne, UK

**Keywords:** ageing, older adults, frailty, pre-frailty, vision impairment

## Abstract

**Background:**

Little is known about vision impairment and frailty in older age. We investigated the relationship of poor vision and incident prefrailty and frailty.

**Methods:**

Cross-sectional and longitudinal analyses with 4-year follow-up of 2836 English community-dwellers aged ≥60 years. Vision impairment was defined as poor self-reported vision. A score of 0 out of the 5 Fried phenotype components was defined as non-frail, 1–2 prefrail and ≥3 as frail. Participants non-frail at baseline were followed-up for incident prefrailty and frailty. Participants prefrail at baseline were followed-up for incident frailty.

**Results:**

49% of participants (n=1396) were non-frail, 42% (n=1178) prefrail and 9% (n=262) frail. At follow-up, there were 367 new cases of prefrailty and frailty among those non-frail at baseline, and 133 new cases of frailty among those prefrail at baseline. In cross-sectional analysis, vision impairment was associated with frailty (age-adjustedandsex-adjusted OR 2.53, 95% CI 1.95 to 3.30). The association remained after further adjustment for wealth, education, cardiovascular disease, diabetes, falls, cognition and depression. In longitudinal analysis, compared with non-frail participants with no vision impairment, non-frail participants with vision impairment had twofold increased risks of prefrailty or frailty at follow-up (OR 2.07, 95% CI 1.32 to 3.24). The association remained after further adjustment. Prefrail participants with vision impairment did not have greater risks of becoming frail at follow-up.

**Conclusion:**

Non-frail older adults who experience poor vision have increased risks of becoming prefrail and frail over 4 years. This is of public health importance as both vision impairment and frailty affect a large number of older adults.

## Introduction

Vision impairment is common in later life.^1^ The most prevalent eye conditions in older age are age-related macular degeneration and cataract, affecting 53% and 36% of British adults aged ≥75 years, respectively.^2^ The rapid growth in the number of older adults in the UK poses a significant public health challenge to improve the health of the older population.^3^ A particular concern in the older population is the development of frailty, estimated to affect between 4% and 17% of adults aged ≥65 years, depending on how it is measured.^4^ Frailty is characterised by an ageing-associated decline in multiple physiological systems reducing the body’s reserve and functional capacity, increasing the vulnerability to adverse outcomes including falls, hospitalisation, institutionalisation and mortality.^5–8^ Frailty is often regarded as a dynamic state along a continuum ranging from normal ageing to death,^9^ and older adults transition between frailty states.^10^ Prefrailty is an intermediate stage between being non-frail and frail,^7^ and the transition towards frailty often ensues from an acute medical event or psychological stress.^10 11^

Vision impairment in older age has been associated with increased risks of adverse health outcomes including functional decline,^12 13^ but little is known about the relationship between vision impairment and frailty. Previous cross-sectional research has shown an association between visual acuity, contrast sensitivity, cataract and frailty indicators including gait speed, grip strength and chair stands in middle-aged and older adults.^14 15^ Cross-sectional analyses of community-dwelling older adults have also showed an association between cataract and Fried frailty phenotype.^16^ However, few studies have investigated incidence of prefrailty as well as frailty, and explored the role of possible mediators such as depression and social isolation, associated with vision impairment and increased risks of frailty.^17 18^ Therefore, we examined the relationship of poor vision with the risk of incident prefrailty and frailty over 4 years adjusting for confounders and exploring possible mediators in a nationally representative sample of community-dwelling English men and women aged ≥60 years.

## Methods

### Study design and participants

In this study, data from the English Longitudinal Study of Ageing (ELSA) was used. ELSA is a prospective study of a nationally representative sample of men and women aged ≥50 years drawn from the Health Survey for England in 1998, 1999 or 2001.^19^ Participants have been followed-up every 2 years for an interview on health and lifestyle and every 4 years from 2004 participants have also been invited to a physical examination. In the present study, we included participants with data on vision impairment, covariates and the Fried phenotype in 2004 and on frailty in 2008. Participants aged <60 years in 2004 were furthermore excluded as the frailty component walking speed was only assessed in participants aged ≥60 years. This generated a sample of 2836 women and men aged ≥60 years (67% of original study sample) used for our analyses. All participants provided informed consent and ethical approval for ELSA was obtained from the Multicentre Research and Ethics Committee.

### Vision impairment

Vision impairment was assessed by asking participants whether their eyesight was excellent, very good, good, fair or poor using glasses or corrective lens if they normally do so. Good vision was defined as reporting excellent, very good or good eyesight and was used as the reference group. Reporting fair or poor eyesight was classified as poor vision. The question on self-experienced eyesight has previously demonstrated a significant association with objectively measured eyesight.^20^

### Assessment of frailty

Participants’ frailty status was first assessed in 2004 and then again in 2008, which allowed for participants to be followed-up for 4 years. Frailty was based on the five components of the Fried phenotype: weight loss, weak grip strength, slow walking, exhaustion and low physical activity.^7^ Identical or very similar definitions of the components to those in the original phenotype studies were operationalised.^7 21^ Weight loss was defined as either loss of ≥10% of body weight in the last 4 years or current body mass index (BMI) <18.5 kg/m^2^. Grip strength was assessed using a grip gauche three times for each hand and the maximum handgrip strength measure out of a total of six attempts was used for the analysis. Weak grip strength was classified as being in the lowest quintile of the distribution, after taking sex and BMI into account. Slow walking speed was based on the mean of the time taken to complete an 8-feet walk at their usual pace from two measurements. The lowest sex-specific and height-specific quintile of the study sample distribution, and those in wheelchairs, bed bound, unable to walk due to health problems or unable to walk alone were classified as having slow walking speed. Exhaustion was defined as giving positive responses to any of the two questions “Felt that everything I did was an effort in the last week” or ‘Could not get going in the last week’ from the Center for Epidemiologic Studies Depression Scale (CES-D).^22^ Low physical activity was based on frequency and intensity in exercise by asking participants how often they undertook vigorous, moderate and mild exercise (more than once a week, once a week, one to three times a month, hardly ever or never). Reporting exercising hardly ever or never, doing mild exercise only or doing moderate exercise a maximum of one to three times a month was classified as low physical activity. Frailty was defined as the presence of three or more of the five frailty components. Prefrailty was defined as the presence of one or two components. No prevalent frailty was defined as having none of the frailty components. The Fried phenotype was used because it incorporates both prefrailty and frailty and is a widely used definition suitable for identification of community-dwelling older adults at increased risk of frailty.^23 24^

### Covariates

Variables considered as covariates included age, sex, wealth, education, cardiovascular disease (CVD), smoking, hypertension, diabetes, history of falls, cognitive function, depression and lack of companionship. Age was grouped into 60–69 years, 70–79 years and ≥80 years. Wealth was based on total net non-pension wealth (financial, housing and physical wealth) of the household presented by quintiles. Education was defined as having an intermediate or higher qualification compared with no qualification. Self-reported doctor-diagnosed CVD (myocardial infarction, angina and/or stroke), diabetes and hypertension were analysed dichotomously. History of falls was based on participants reporting fallen down in the last 12 months. Smoking was defined as reporting being a current smoker or current non-smoker. Cognitive function was assessed using a validated 24-point cognitive scale on time orientation (4 points), immediate recall (10 points) and delayed recall (10 points) and analysed continuously.^25^ For the recall tests, participants were presented with a list of 10 words of which they were asked to recall as many words as possible immediately after the list was read, and then again after an approximately 5 min delay during which they completed other survey questions. Data on time orientation were obtained from the Mini Mental Status Examination and assessed by asking participants to report today’s day, date, month and year verbally.^26 27^ Factors that may be on the causal pathway of vision impairment and frailty such as depression and social interaction were also considered. Depression symptoms were based on the six questions on mood not part of the frailty component exhaustion from the validated 8-item version of CES-D.^22^ Reporting two or more items were defined as having depression symptoms and analysed dichotomously. Feeling lack of companionship some of the time or often were combined and compared with feeling no lack of companionship.

### Statistical analyses

Logistic regression was used to determine cross-sectional relationships of vision impairment with prefrailty and frailty combined. Logistic regression was also used to determine the odds of incident prefrailty and frailty combined over 4 years follow-up in non-frail participants at baseline with poor self-reported vision compared with those with good vision. Similarly, we determined the odds of incident frailty in participants prefrail at baseline, followed-up for 4 years. Analyses also included investigating possible reverse relationships in participants prefrail and/or frail at baseline and non-frail at follow-up, and participants frail at baseline and prefrail at follow-up. Good vision was used as the reference group. The statistical analyses were adjusted for age, sex and covariates significantly associated with vision impairment in participants non-frail and prefrail at baseline (wealth, CVD, diabetes, cognitive function, falls) (table 1) and for covariates that have consistently been associated with both vision impairment and frailty in previous research, such as educational level and depression.^7 17 28^ Where a positive association was demonstrated between vision impairment and frailty, supplementary analyses were conducted to explore if the association might be explained by lack of companionship, a marker of social isolation associated with both vision impairment and frailty.^17 18^ All analyses were carried out using SPSS (V.22, IBM, Armonk, New York, USA).

**Table 1:** Age, sex, socioeconomic and lifestyle characteristics, comorbidities and falls and prevalence of non-frailty, prefrailty and frailty in a cohort of English men and women aged 60 years and over in 2004 (baseline)

	Overall	Good vision	Poor vision	p Value
Totals, (n)%	2836 (100)	2497 (88)	339 (12)	
Covariates				
Age in years, (n)%				
60–69	1526 (54)	1400 (56)	126(37)	<0.01
70–79	1012 (36)	865 (35)	147 (43)	
80+	298 (11)	232 (9)	66 (20)	
Male gender, (n)%	1584 (56)	1121 (45)	131 (39)	0.03
Wealth, (n)%				
1 (lowest)	396 (14)	309 (13)	87 (26)	<0.01
2	541 (19)	452 (18)	89 (27)	
3	562 (20)	505 (20)	57 (17)	
4	615 (22)	563 (23)	52 (16)	
5 (highest)	690 (24)	643 (26)	47 (14)	
No educational qualification, (n)%	1086 (38)	915 (37)	171 (50)	<0.01
Smoker, (n)%	308 (11)	250 (10)	58 (17)	<0.01
Body mass index, mean±SD	27.8 (4.6)	27.7 (4.5)	28.3 (4.8)	0.03
Hypertension, (n)%	1302 (46)	1123 (45)	179 (53)	<0.01
Cardiovascular disease, (n)%	499 (18)	400 (16)	99 (29)	<0.01
Diabetes, (n)%	251 (9)	197 (8)	54 (16)	<0.01
Cognitive function, mean±SD	13.7 (3.3)	13.9 (3.3)	12.8 (3.2)	<0.01
Depression symptoms, (n)%	653 (23)	536 (22)	117 (35)	<0.01
History of falls, (n)%	882 (31)	742 (30)	140 (41)	<0.01
*Frailty prevalence at baseline (2004), n(%)*				
Non-frail, n(%)	1396 (49)	1303 (52)	93 (27)	<0.01
Prefrail, n(%)	1178 (42)	1010 (40)	168 (50)	<0.01
Frail, n(%)	262 (9)	184 (7)	78 (23)	<0.01

## Results

In 2004, 2836 adults (56% men) aged ≥60 years completed an interview and a physical examination. Self-reported vision impairment was prevalent in 12% (n=339) of the participants. Half of the participants (n=1396) (49%) had no prevalent frailty, 1178 (42%) were prefrail and 262 (9%) were frail. Those who were prefrail or frail at baseline were excluded to determine incident frailty. The participants were followed for 4 years during which a total of 367 new cases of prefrailty (n=343) and frailty (n=24) among those without prevalent frailty at baseline were reported. In addition, there were 133 new cases of frailty in participants prefrail at baseline.

### Cross-sectional associations of vision impairment and frailty at baseline

Table 1 presents the characteristics of all participants in 2004 (baseline) by vision impairment. In comparison to participants with good vision, those with poor vision were more likely to be older, male gender, less wealthy, no educational qualification, smoker, higher BMI, diagnosed with hypertension, CVD and diabetes, a history of falls, poorer cognitive function and depression symptoms. Half of older adults with poor vision (50%, n=168) were prefrail and 23% (n=78) were frail, in comparison 40% (n=1010) of older adults with good vision were prefrail and 7% (n=184) were frail.

OR with 95% CI for the cross-sectional associations between vision impairment and frailty are presented in table 2. The cross-sectional associations between vision impairment and frailty at baseline showed that participants with poor vision had over twofold greater odds of being prefrail or frail compared with participants with good vision (age-adjusted and sex-adjusted OR 2.53, 95% CI 1.95 to 3.30) and the association remained after further adjustment for wealth, education, CVD, diabetes, falls, cognition and depression (OR 1.72, 95% CI 1.30 to 2.29). The association remained after additional adjustment for lack of companionship in a subsample of 2663 participants with such data (OR 1.65, 95% CI 1.22 to 2.22). Cross-sectional analyses of the relationships between vision impairment and prefrailty and frailty separately showed that both prefrailty (OR 2.10, 95% CI 1.59 to 2.77) and frailty (OR 4.83, 95% CI 3.30 to 7.06) were statistically significantly associated with vision impairment (see online supplementary appendix table S1). In supplementary analyses on individual, frailty components vision impairment was associated with exhaustion, low physical activity and slow gait speed on adjustment for multiple covariates suggesting the associations observed were not driven by a single frailty component. Vision impairment was also associated with age-adjusted and sex-adjusted odds for weak grip but attenuated after further adjustment for wealth and education. Weight loss was not associated with vision impairment (see online supplementary appendix table S2). There was no interaction between vision impairment and gender.

10.1136/jech-2017-209207.supp1 Supplementary file 1

**Table 2:** ORs with 95% CIs for cross-sectional associations between frailty (prefrailty and frailty combined) and vision impairment in English men and women aged 60 years and over in 2004

	Good vision	Poor vision
Participants with prefrailty and frailty combined, n (%)	1194 (48)	246 (73)
Models for adjustment	OR	OR (95% CI)
Age-adjusted and sex-adjusted	1.00	2.53 (1.95–3.30)
Multi-adjusted*	1.00	1.72 (1.30–2.29)

*Multi-adjusted=adjusted for age, sex, wealth, education, cardiovascular disease, diabetes, falls, cognition, depression.

### Longitudinal associations of vision impairment with incident prefrailty and frailty

Longitudinal analyses were carried out in non-frail participants at baseline (excluding participants with prefrailty and frailty) and participants with prefrailty at baseline. Table 3 presents the characteristics of participants who were non-frail and prefrail, respectively, at baseline by vision impairment. In non-frail participants, poor vision was associated with advanced age, lower wealth, no educational qualification, CVD and lower cognitive function. Participants prefrail at baseline with poor vision were associated with advanced age, lower wealth, CVD, diabetes, lower cognitive function and falls.

**Table 3:** Age, sex, socioeconomic and lifestyle characteristics, comorbidities and falls in a cohort of English men and women aged 60 years and over with no frailty and with prefrailty, respectively, in 2004 (baseline)

	1396 participants with no frailty in 2004				1178 participants prefrail in 2004			
	Overall	Good vision	Poor vision	p Value	Overall	Good vision	Poor vision	p Value
Totals, n (%)	1396 (100)	1303 (93)	93 (7)		1178 (100)	1010 (86)	168 (14)	
Age in years, n (%)								
60–69	864 (62)	827 (64)	37 (40)	<0.01	568 (48)	502 (50)	66 (39)	<0.01
70–79	459 (33)	415 (32)	44 (47)		455 (39)	388 (38)	67 (40)	
80+	73 (5)	61 (5)	12 (13)		155 (13)	120 (12)	35 (21)	
Male gender, n (%)	785 (56)	734 (56)	51 (55)	0.78	413 (35)	348 (35)	65 (39)	0.29
Wealth, n (%)								
1 (lowest)	112 (8)	94 (7)	18 (20)	<0.01	195 (17)	158 (16)	37 (23)	<0.01
2	204 (15)	191 (15)	13 (14)		264 (22)	215 (21)	49 (30)	
3	278 (20)	262 (20)	16 (18)		242 (21)	207 (21)	35 (22)	
4	346 (25)	326 (25)	20 (22)		236 (20)	212 (21)	24 (15)	
5 (highest)	436 (31)	412 (32)	24 (26)		230 (20)	212 (21)	18 (11)	
No education, n (%)	420 (30)	383 (29)	37 (40)	0.04	507 (43)	426 (42)	81 (48)	0.14
Smoker, n (%)	103 (7)	94 (7)	9 (10)	0.38	162 (14)	133 (13)	29 (17)	0.15
Body mass index, mean±SD	27.2 (3.9)	27.2 (3.9)	27.1 (4.4)	0.70	27.9 (4.8)	27.9 (4.8)	28.3 (4.7)	0.32
Hypertension, n (%)	560 (40)	517 (40)	43 (46)	0.21	583 (50)	491 (49)	92 (55)	0.14
Cardiovascular disease, n (%)	195 (14)	173 (13)	22 (24)	0.01	213 (18)	172 (17)	41 (24)	0.02
Diabetes, n (%)	90 (6)	80 (6)	10 (11)	0.08	121 (10)	92 (9)	29 (17)	<0.01
Cognitive function, mean±SD	14.2 (3.3)	14.3 (3.1)	13.5 (3.2)	0.03	13.7 (3.4)	13.6 (3.4)	12.8 (3.2)	<0.01
Depression symptoms, n (%)	149 (11)	136 (11)	13 (14)	0.29	362 (31)	302 (30)	60 (36)	0.11
History of falls, n (%)	347 (25)	320 (25)	27 (29)	0.34	396 (34)	326 (32)	70 (42)	0.02

Table 4 shows OR with 95% CIs for incident prefrailty and frailty in participants non-frail at baseline, and incident frailty in those prefrail at baseline. Among participants non-frail at baseline, those who reported poor vision had a twofold increased risk of becoming prefrail or frail at 4-year follow-up (age-adjusted and sex-adjusted OR 2.07, 95% CI 1.32 to 3.24) compared with participants with good vision. The association remained after further adjustment for wealth, education, CVD, diabetes, falls, cognition and depression. Additional analysis was carried out in a subsample of 1338 non-frail participants with data on lack of companionship. In this analysis, vision impairment remained associated with increased risks of prefrailty and frailty after further adjustment for lack of companionship (OR 1.98, 95% CI 1.23 to 3.19). Vision impairment was not associated with an increased risk of frailty in older adults prefrail at baseline (OR 1.34, 95% CI 0.82 to 2.19). Analyses of possible reverse relationships showed no associations after adjustment for covariates.

**Table 4:** ORs with 95% CIs for associations between incidence of prefrailty and frailty with vision impairment in English men and women aged 60 years and over in 2004 followed-up for 4 years to 2008

	Good vision	Poor vision
No prevalent frailty at baseline	n=1303	n=93
Prefrail or frail at follow-up, n (%)	324 (25)	43 (46)
Models for adjustment	OR	OR (95% CI)
Age-adjusted and sex-adjusted	1.00	2.07 (1.32–3.24)
Multi-adjusted*	1.00	1.86 (1.17–2.95)
Prefrail at baseline	n=1010	n=168
Frail at follow-up, n (%)	107 (11)	26 (16)
Models for adjustment	OR	OR (95% CI)
Age-adjusted and sex-adjusted	1.00	1.34 (0.82–2.19)

*Multi-adjusted=adjusted for age, sex, wealth, education, cardiovascular disease, diabetes, falls, cognition, depression.

## Discussion

In this study, we examined the association of vision impairment with incident frailty in older age. Our findings show that non-frail older adults with self-reported poor vision have increased risks of becoming prefrail or frail compared with non-frail older adults with good vision. Vision impairment, however, was not significantly associated with incident frailty in those already prefrail at baseline. This is to our knowledge the first study investigating the relationship between vision impairment and incident frailty using the Fried phenotype.

The longitudinal analyses showed that non-frail older adults with vision impairment had nearly twice the risk of becoming prefrail and frail compared with those with good vision on adjustment for covariates. This is consistent with earlier cross-sectional population-based studies of middle-aged and older community-dwelling adults investigating the association between objectively measured vision impairment^14^ and cataract,^15^ respectively, and frailty assessed using a frailty score consisting of slow gait speed, low expiratory flow rate, poor handgrip strength and inability to perform chair stands. In contrast, prefrail participants with vision impairment did not have an increased risk of developing frailty. This is similar to a previous cross-sectional study on vision problems defined as having cataracts and frailty defined as the Fried phenotype reporting that cataract was associated with prefrailty but not with frailty.^16^ This finding suggests that vision impairment may be of particular importance in the onset of the early stages of frailty, rather than progression in those already prefrail.

Several factors could explain the longitudinal relationship observed. Vision impairment is associated with a range of comorbidities known to be associated with frailty such as CVD and diabetes.^29^ In our study, the associations remained after adjustment for several comorbidities including diabetes, CVD and depression, and while some residual confounding is possible due to lack of adjustment of potential confounding factors, the relationship observed could also be explained by factors on the causal pathway between vision impairment and frailty such as social isolation. Being socially engaged may reduce the impact of loss of physiologic reserve associated with frailty.^18^ Vision impairment has also been associated with being socially isolated,^17^ and vision impaired older adults may not therefore be able to benefit from the positive effects of social support in preventing frailty. Our supplementary analysis of a subsample with data on companionship suggests that this does not fully explain the relationship observed; however, we had insufficient data to also explore the role of social activities and social networks. Finally, it is possible that shared pathological pathways such as inflammation, which has been associated with both vision impairment and frailty,^30 31^ may in part explain the association observed.

### Strengths and limitations

A major strength of the study is that data are from a nationally representative cohort of community-dwelling English women and men aged ≥60 years.^32^ Also, participants were followed-up for 4 years for prefrailty and frailty, and the models were adjusted for several important confounding factors. Limitations include that vision impairment was self-reported rather than objectively measured. However, the question used has been against objective measures,^20^ and the finding is comparable to national estimates.^1^ In this study, a slightly modified version of the validated Fried phenotype was used due to limitations in the data. Levels of physical activity referred to frequency and intensity of exercise without information on calorie consumption and the ELSA data did not allow differentiating between intentional and unintentional weight loss. However, the data used were obtained through an interview and physical examination and the prevalence of frailty in this study is comparable to the original Fried phenotype study.^7^ Another limitation is that our study was restricted to the two-thirds of ELSA participants with data on frailty measurements at both baseline and follow-up. Of 5918 participants aged *≥*60 years in 2004, 1670 were lost to follow-up in 2008. Non-respondents were more likely to be older and have poorer health compared with respondents. This raises potential selection bias, suggesting that prevalence of vision impairment, prefrailty and frailty might have been higher among non-respondents. Furthermore, in our study vision impairment was measured at baseline only and we did not investigate the primary cause of or change in vision impairment. Finally, the ELSA cohort comprised predominantly of white British people and the findings may not be generalisable to other ethnic groups.

### Implications

The association observed between vision impairment in non-frail older adults and increased risks of becoming prefrail and frail is important from a public health perspective as both vision impairment and frailty affect a large number of people in later life.^1 4^ Vision impairment is often preventable and modifiable.^33^ For instance, a healthy lifestyle may reduce the risks of developing macular degeneration. Also, cataract surgery can significantly improve visual functioning.^34^ The findings of this study support previous research suggesting that early diagnosis and treatment of vision impairment could prevent non-frail older adults with vision impairment to enter the early stage of frailty.^16^ Reducing the risk of frailty is essential as frailty decreases the chances of independent living, negatively affecting the health and well-being of the individual and increasing the financial costs of healthcare to society.^7 35 36^

## Conclusions

This study shows that self-reported poor vision is associated with the onset of prefrailty and frailty in later life. Preventing and treating vision impairment in later life may have the potential to delay the development of frailty.

**What is already known on this subject**

Vision impairment is common in later life and associated with a range of adverse health outcomes including comorbidities, physical disability and poor social interaction affecting overall well-being and independent living. Vision impairment may furthermore play a role in the development of frailty, another common health concern in older age. However, evidence on the prospective relationship between vision impairment and development of frailty is sparse.

**What this study adds**

Our findings show that compared with non-frail participants with no vision impairment, non-frail participants with vision impairment had a twofold increased risk of becoming prefrail or frail at follow-up and the association remained after further adjustment for wealth, education, cardiovascular disease, diabetes, falls, cognition and depression. These findings suggest that vision impairment in older age contributes towards the development of frailty. Given the rapidly increasing number of older adults, addressing age-related vision problems may be important to prevent older adults from becoming frail, which further affects the chances of functional independence in later life.
