# Supplementary material for: Self-reported vision impairment and incident prefrailty and frailty in English community-dwelling older adults: findings from a 4-year follow-up study
Source: J Epidemiol Community Health. 2017 Aug 10;71(11):1053–8. doi: 10.1136/jech-2017-209207 (PMC5847099; doi:10.1136/jech-2017-209207)
Supplement: Supplementary file 1 [file jech-2017-209207supp001.pdf]

Appendix. Table S1. Odds ratios (OR) with 95% CIs for cross-sectional associations between pre-frailty and frailty, separately, and vision impairment in English men and women aged 60 years and over in 2004

	Good vision	Poor vision
Participants with pre-frailty, n (%)	1010 (40)	168 (50)
Models for adjustment	OR	OR (95% CI)
Age- and sex-adjusted	1.00	2.10 (1.59-2.77)
Multi-adjusted*	1.00	1.56 (1.16-2.10)
Participants with frailty, n (%)	184 (7)	78 (23)
Models for adjustment	OR	OR (95% CI)
Age- and sex-adjusted	1.00	4.83 (3.30-7.06)
Multi-adjusted*	1.00	2.88 (1.83-4.54)

*Multi-adjusted=adjusted for age, sex, wealth, education, CVD, diabetes, falls, cognition, depression

Appendix. Table S2. Odds ratios (OR) with 95% CIs for cross-sectional associations between individual frailty components and vision impairment in English men and women aged 60 years and over in 2004

Individual frailty components	Good vision	Poor vision
Models for adjustment	OR	OR (95% CI)
Exhaustion, n (%)	558 (22)	142 (42)
Age- and sex-adjusted	1.00	2.34 (1.84-2.97)
Model 2 (M2): M1 + wealth and education	1.00	2.14 (1.67-2.73)
Model 3 (M3): M2 + CVD and diabetes	1.00	1.99 (1.56-2.56)
Model 4 (M4): M3 + falls	1.00	1.94 (1.51-2.49)
Model 5 (M5): M4 + cognition	1.00	1.90 (1.48-2.45)
Multi-adjusted*	1.00	1.81 (1.37-2.39)
Low physical activity, n (%)	363 (15)	121 (36)
Age- and sex-adjusted	1.00	2.90 (2.25-3.74)
Multi-adjusted*	1.00	2.03 (1.55-2.67)
Slow gait speed, n (%)	440 (18)	131 (39)
Age- and sex-adjusted	1.00	2.48 (1.93-3.19)
Multi-adjusted*	1.00	1.71 (1.31-2.24)
Weak grip, n (%)	414 (17)	96 (28)
Age- and sex-adjusted	1.00	1.51 (1.11-2.05)
Adjusted for age, sex, wealth and education	1.00	1.30 (0.95-1.79)
Weight loss, n (%)	136 (5)	26 (8)
Age- and sex-adjusted	1.00	1.49 (0.95-2.32)

*Multi-adjusted=adjusted for age, sex, wealth, education, CVD, diabetes, falls, cognition, depression
